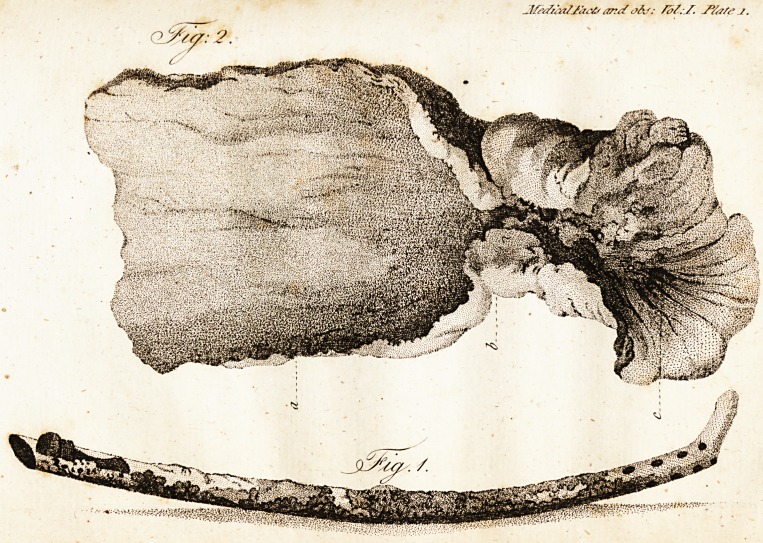# Case of a Catheter, Left in the Bladder, in Drawing off the Urine, for a Retroversion of the Uterus

**Published:** 1791

**Authors:** Edward Ford

**Affiliations:** Surgeon of the Westminster General Dispensary.


IX. *Cafe of a Catheter, left in the Bladder, in drawing off the Urine, for a retroverfion of the Uterus. By Mr.* Edward Ford, *Surgeon of the Weftminfter General Difpenfary.*

MARY WILDING, a thin delicate woman, about twenty-five years of age, was admitted in January laft, as a patient at the Weftminfter General Difpenfary. She complained plained of a painful and involuntary diſcharge of urine, mixed with blood and matter, from the urethra; and alſo of a diſcharge of purulent urine, which was continually flowing from a fiſtulous ſore, ſituated in the buttock, near the middle of the glutæus muſcle. She was in a weak and emaciated ſtate, and had been confined to her bed for ſeveral months; every attempt to move from thence being attended with moſt ſevere pains, both in the neck of the bladder, and at the fiſtulous wound in the nates.

Upon introducing a ſound into the bladder, an extraneous ſubſtance was eaſily felt within its cavity; and from its hardneſs, I judged that it might be a calculous concretion. At the patient's deſire, I then proceeded to examine the fiſtulous ſore on the buttock, and ſhe told me there was a looſe bit of bone in the wound, which frequently made its way outwards beyond the ſkin, but as often ſeemed to be retracted with conſiderable force. I found by examining it with the probe that it lay looſe in the ſinus, and I endeavoured to remove it with the forceps, gradually drawing it outwards. This proceſs was not attended with much pain; but when the extraneous ſubſtance was brought forward about half an inch beyond the integuments, a further removal of it feemed impracticable, as it was ftrongly held back by the contraction of the mufcles. Whilft it was retained externally by the forceps, I viewed it clofely to afcertain whether it was an exfoliation of carious bone, or a calculous concretion that had made its way outwards from the bladder, but was much aftonifhed to find that the fubftance protruded from the wound, was evidently the bulbous end of a filver catheter.

This difcovery inftantly induced me to fufpend any further operation, as it was clear that an attempt to remove the catheter by extracting it forcibly through the wound, muft occafion a confiderable laceration of the fundus of the bladder; and I was anxious to collect from the patient, fuch circumftances, as might explain her unfortunate fituation. She profeffed herfelf totally ignorant by what means the catheter had been lodged in her bladder, and could with difficulty believe the information I gave her. The narrative fhe furnifhed me with was, that fhe had been brought to bed four months; that in the third month of her laft pregnancy, fhe had been feized with a difficulty of voiding her urine, which had been feveral times drawn off by means of a catheter; that fhe had experienced great relief from this operation, but that the laſt time it was performed ſhe had felt great pain, and had ever ſince been unable to remove from her bed without great diſtreſs; that ſhe had been ſafely delivered at the expiration of the ninth month; and that ſhe had ſince ſuckled her infant, though in the moſt wretched and debilitated ſtate. It was obvious from this account, that the catheter had eſcaped from the hands of the operator the laſt time the urine had been drawn off; that it had ſlipped into the bladder, and had been ſuffered to remain there; and that the only method of relieving her was to extract it through the meatus urinarius.

From the weak ſtate in which ſhe lay, exhauſted by ſuckling her infant, by pain, and by the diſcharge from the wound, I declined performing the operation till her health ſhould be a little invigorated by weaning the child, and by a more nouriſhing diet. When this was accompliſhed, I was favoured with the aſſiſtance of Dr. Jackſon, Dr. Bland, and Dr. Combe, all of whom were anxious to ſee ſo ſingular a caſe.

The patient was laid upon a table and ſecured in the manner uſually adopted in the operation of Lithotomy. The urethra was dilated by the blunt gorget introduced upon a female ſtaff, and the catheter was then attempted to be taken out by the forceps. This part of the operation was attended with much difficulty, as the catheter lay tranfverfely in the bladder, the handle of it refting on the arch of the pubis, and its other extremity on the crura ifchii. It was diflodged from its fituation by drawing the blunt end outwards through the pofterior wound, fo that the handle of the inftrument being detached from the pubes, was more eafily brought forward through the opening in the urethra, and extracted. The catheter, which is now in my poffeffion, was found covered with a flight incruftation, as reprefented in the annexed drawing *. The operation was finifhed by extracting a few fmall calculi from the bladder. The patient was then put to bed, and the fame regimen purfued as after cutting for the ftone. A flight fever came on, but was apparently more owing to the ftate of her breafts, as fhe had juft weaned her child, than to the operation. The fiftulous opening on the buttock healed in a few days, the urine paffing entirely through the natural paffage; and in one month fhe was perfectly well. She now retains her urine, and fuffers no inconvenience from this extraordinary calamity.

The foregoing cafe is, I believe, un-

* See plate I., fig. 1.

precedented

Fig: 2.

Fig. 1.

precedented in medical hiſtory. It affords a ſingular example of an accident occurring from an operation in ſurgery, which has uſually been deemed eaſy to perform, and free from hazard. The natural ſtructure and ſituation of the female urethra warrants the general opinion of the ſafety of this operation; but when an alteration takes place in theſe parts, either from pregnancy or other cauſes, the operation of drawing off the urine may become liable to difficulty.

In caſes of retroverted uterus, we find, by the teſtimony of Dr. Hunter, and other practitioners, that this operation is not always to be done with facility, and that in ſome caſes it has been impracticable. The poor woman who is the ſubject of this paper had been liable to a retroverſion of the womb, both in this and in a former pregnancy. Her urine had been drawn off a few days before this accident by a man-midwife of eminence; but being ſuddenly taken ill, ſhe applied to a perſon in her neighbourhood, from whom this accident happened. His buſineſs obliging him inſtantly to leave London, he heard no more of his patient, and imagined, I ſuppoſe, that the catheter had been expelled by the efforts of the bladder.

*Golden Square,*
May 16, 1791.

## Figures and Tables

**Fig. 1. Fig: 2. f1:**